# Dynamic Hydrogels against Infections: From Design to Applications

**DOI:** 10.3390/gels10050331

**Published:** 2024-05-14

**Authors:** Ming Zhang, Chongyu Zhu

**Affiliations:** College of Chemistry and Chemical Engineering, Donghua University, Shanghai 201620, China; zming_0613@163.com

**Keywords:** responsive hydrogels, dynamic chemical bonds, antimicrobial and antiviral treatments, controlled drug release, self-healing, inherent anti-infection performance

## Abstract

Human defense against infection remains a global topic. In addition to developing novel anti-infection drugs, therapeutic drug delivery strategies are also crucial to achieving a higher efficacy and lower toxicity of these drugs for treatment. The application of hydrogels has been proven to be an effective localized drug delivery approach to treating infections without generating significant systemic adverse effects. The recent emerging dynamic hydrogels further show power as injectable formulations, giving new tools for clinical treatments. In this review, we delve into the potential applications of dynamic hydrogels in antibacterial and antiviral treatments and elaborate on their molecular designs and practical implementations. By outlining the chemical designs underlying these hydrogels, we discuss how the choice of dynamic chemical bonds affects their stimulus responsiveness, self-healing capabilities, and mechanical properties. Afterwards, we focus on how to endow dynamic hydrogels with anti-infection properties. By comparing different drug-loading methods, we highlight the advantages of dynamic chemical bonds in achieving sustained and controlled drug release. Moreover, we also include the design principles and uses of hydrogels that possess inherent anti-infective properties. Furthermore, we explore the design principles and applications of hydrogels with inherent anti-infective properties. Finally, we briefly summarize the current challenges faced by dynamic hydrogels and present a forward-looking vision for their future development. Through this review, we expect to draw more attention to these therapeutic strategies among scientists working with chemistry, materials, as well as pharmaceutics.

## 1. Introduction to Dynamic Hydrogels

Hydrogels are polymeric materials with three-dimensional (3D) network structures [1], renowned for their unique water-retaining properties. Due to their high biocompatibility and ease of manufacturing, hydrogels have found diverse applications in agriculture [2], cell engineering [3], the food industry [4], and, particularly, biomedicine. For instance, common infections caused by bacteria, fungi, and viruses pose a significant threat to human health [5,6]. Despite the availability of various anti-infection agents, people are still concerned about their potential adverse effects and limited efficacy, especially when it comes to systemic treatment for localized infections. With the aid of hydrogels, these anti-infection drugs can be encapsulated and precisely delivered to the infected sites, resulting in an enhanced efficacy and minimized toxicity to the human body. Furthermore, serving as wound dressings, hydrogels help maintain wound moisture, promote the healing process, and protect it against further infections or damages [7,8].

Compared to conventional hydrogels, the recently developed dynamic hydrogels emerge as a superior option for wound care due to their unique adaptive properties. Dynamic hydrogels are hydrogels that contain dynamic chemical bonds in their 3D networks. These internal dynamic bonds will break and reform under certain conditions, granting them a self-healing performance to provide durable protection for wounds. The dynamic feature also allows for the design of injectable hydrogels, enriching the administration forms for anti-infections [9]. Additionally, by leveraging the dynamic nature of these chemical bonds, researchers can introduce anti-infection agents into hydrogels through bonding in addition to encapsulation, thereby enhancing drug encapsulation efficiency and achieving the controlled and on-demand release of these agents. This approach will not only provide more precise therapy but also improve the overall efficacy of wound care, facilitating faster healing and minimizing the risk of infection [10].

Therefore, in this mini review, we aim to summarize the recent achievement of dynamic hydrogels and their applications in anti-infection treatment. We will introduce the chemical design of these hydrogels, emphasizing their unique dynamic properties and their role in enhancing the overall efficacy of anti-infection treatment. We expect to gain a better understanding of the potential applications of these dynamic hydrogels in anti-infection therapy and their contributions to advancements in medical technology. Additionally, we will offer our perspectives on the future trajectory of dynamic hydrogels in anti-infection treatment, speculating on how they might revolutionize this critical area of medical research and practice.

## 2. Chemical Design of Dynamic Hydrogels

The key to the design of dynamic hydrogels relies on the selection of appropriate dynamic chemical bonds [11]. Despite the availability of numerous types of dynamic chemical bonds, imine bonds, boronic ester bonds, and disulfide bonds stand out as the most frequently chosen options for constructing dynamic hydrogels, due to the considerations of the bio-safety and widespread accessibility of the raw materials [12]. Therefore, in this section, we aim to provide a brief overview of the role dynamic bonds play in the fabrication of dynamic hydrogels. We will mainly discuss the utilization of different dynamic bonds, emphasizing their distinct properties and exploring the relationship between the bond type and hydrogel characteristics.

**Figure 1:**
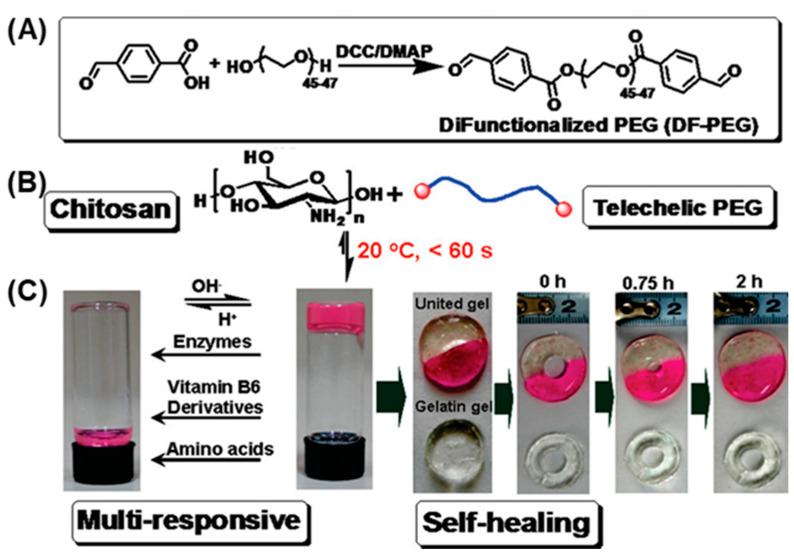
Investigation into the preparation and properties of dynamic imine bond hydrogels. (**A**) Synthesis of DF-PEGs; (**B**) DF-PEGs coupled with chitosan to form dynamic hydrogels; (**C**) The multi-responsive and self-healing properties of dynamic hydrogels. Reprinted with permission from Ref. [15]. Copyright © 2011 American Chemical Society.

The imine bond is an important type of dynamic chemical bond formed through the condensation reaction between amino groups and aldehyde or ketone groups. When environmental conditions change, these imine bonds undergo a variety of reactions, including amino transfer, hydrolysis, and imine bond metathesis [13,14]. Consequently, they can be applied to construct dynamic hydrogels with self-healing properties. For example, Tao and Wei synthesized a dialdehyde-functionalized polyethylene glycol (DF-PEG) (Figure 1A), which reacted rapidly with the amino groups in chitosan via a Schiff base reaction, yielding a self-healing hydrogel containing dynamic imine bonds [15] (Figure 1B). Owing to the spontaneous breakage and reformation of the imine bonds within the hydrogels, this hydrogel was able to restore into a cohesive, united structure in 2 h, even after being punched with a hole in its center. This result demonstrates that dynamic hydrogels have superior resistance to external damage compared to conventional hydrogels, which is beneficial for the wound-healing process. The dynamic imine bonds also allow this hydrogel to respond to multiple stimuli, including pH vitamin B6 derivatives and the amino acid lysine. In addition, the authors demonstrated the efficient encapsulation and release of lysozyme through this hydrogel, highlighting its potential as a controlled drug release system (Figure 1C). It is worth noting that, given its chitosan-based composition, the hydrogel is susceptible to hydrolysis upon exposure to papain, leading to its structural decomposition, indicating its degradable nature in physiological systems.

**Figure 2:**
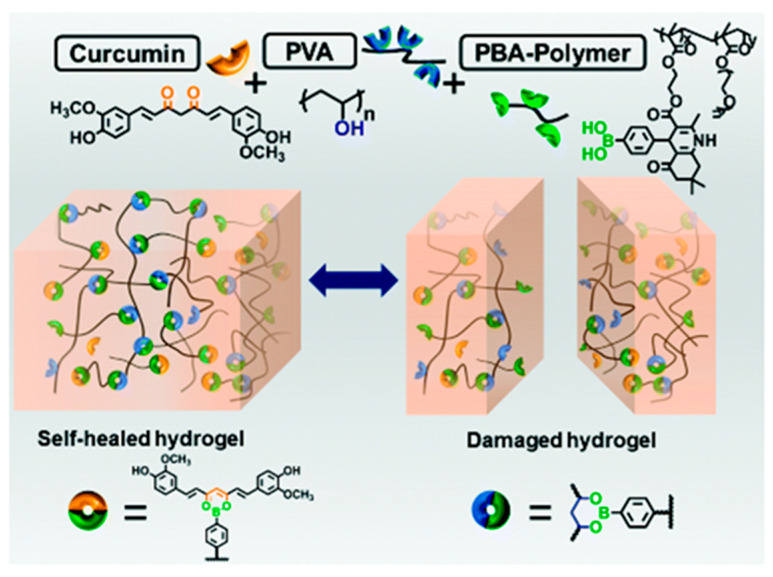
A curcumin-loaded self-healing hydrogel based on the formation of dynamic boronic esters. Reprinted with permission from Ref. [20]. Copyright © 2010 Royal Society of Chemistry.

The boronic ester bond, formed through the condensation reaction between boronic acid groups and molecules containing 1,2-diol or 1,3-diol structures [16,17,18], serves as another crucial chemical bond for constructing dynamic hydrogels. Similar to imine bonds, boronic ester bonds are responsive to changes in pH [19]. Moreover, they can also interact with sugars and hydrogen peroxide (H_2_O_2_), expanding the design for dynamic hydrogels. Despite the availability of various boric acid and polyol sources for the preparation of dynamic hydrogels based on boronic ester bonds, polyvinyl alcohol (PVA) remains a preferred and widely used polyol material due to its low cost and excellent biocompatibility. For instance, Tao’s team prepared a polymer with a phenylboronic acid structure on its side groups using the Hantzsch reaction [20]. By mixing this polymer with PVA at room temperature, they constructed a biocompatible dynamic hydrogel with self-healing and injectable properties. With the aid of phenylboronic acids present in this dynamic hydrogel, they successfully loaded the poorly water-soluble curcumin by forming dynamic boronic ester bonds with its 1,3-diketone group. Furthermore, this drug-loaded hydrogel responded to various stimuli, such as acids and sugars, with a notably rapid curcumin release in the presence of H_2_O_2_, which also induced gel degradation, accelerating the wound healing (Figure 2).

Disulfide bonds are prevalent in proteins and many commercial raw materials. They are cleavable in response to light or reductants and are also exchangeable with thiols under suitable temperature and pH conditions [21,22]. Therefore, they are often used in the construction of dynamic hydrogels. For instance, Yang et al. prepared an injectable dynamic hydrogel using chloroauric acid solution (HAuCl_4_) and keratin, a protein rich in cysteine derived from chicken feathers [23]. During the mixing of the two solutions, the cysteine thiol groups in keratin reduced Au (III) to Au (I), while the Au (I) then reacted with the thiol groups to form Au-S bonds, acting as crosslinking netpoints for the hydrogel. Thanks to the dynamic exchange reactions between the Au-S bonds and disulfide bonds, this hydrogel can not only be injected and fit the wound site under physiological pH conditions but also be biodegradable in vivo. Without the additional chemical modification of the keratin, this hydrogel exhibits a good hemostatic effect. 

Compared to single dynamic chemical bonds, hydrogels with dual dynamic chemical bonds exhibit higher controllability and tunability [24,25]. For example, Zhang et al. designed a multifunctional boronic acid-based crosslinker, bis (phenylboronic acid carbamoyl) cystamine (BPBAC), to construct dynamic hydrogels featuring both boronic ester and disulfide bonds [26]. The boronic ester bonds in these hydrogels are responsive to both pH and glucose, while the disulfide bonds confer them a redox response, respectively. These multi-responsive hydrogels offer a feasible solution for the precise control of the network of the hydrogels and their mechanical properties. Wang et al. introduced both dynamic imine bonds and boronic ester bonds to prepare a self-healing PVA/PEI/DCMC/CMCS hydrogel with antibacterial properties [27]. The aldehyde groups in dialdehyde carboxymethyl cellulose (DCMC) were reacted with the amine groups in polyethyleneimine (PEI) and carboxymethyl chitosan (CMCS) to form dynamic imine bonds. Borax was used as a crosslinking agent to react with the hydroxyl groups in polyvinyl alcohol (PVA) and form borate ester bonds. Possessing double chemical crosslinking, this hydrogel was self-healable at room temperature and exhibits excellent mechanical properties with an elongation at a break of up to 1025%.

## 3. Dynamic Hydrogels for Anti-Infection Applications

Bacteria, fungi, and viruses are common sources of infection that can cause various diseases, posing a serious threat to human health [28,29,30]. To treat these infections, scientists are dedicated to developing targeted drugs. However, concerns have been raised regarding overdosages and incorrect administration methods, as they can compromise drug efficacy, promote infection resistance, and even result in human poisoning or fatalities [31,32,33]. 

Fortunately, hydrogels-based drug delivery has emerged as a promising approach for treating local infections [34]. By applying hydrogels to wounds, infections can be efficiently suppressed with minimal side effects, enhancing the therapeutic experience for patients. Currently, there are two major categories of anti-infection dynamic hydrogels: drug-loaded and inherent. Within the drug-loaded hydrogels, the anti-infection agents can be incorporated using various methods, resulting in drug-encapsulated or drug-bonded hydrogels.

Drug-encapsulated hydrogels involve the direct encapsulation of anti-infection agents within the interior of the dynamic hydrogels. While the preparation of these hydrogels is straightforward, the absence of a chemical interaction between the drug and the hydrogel network often poses challenges. Specifically, the efficiency of the drug loading and release profile can vary significantly depending on the hydrophilic or hydrophobic nature of the drug [35].

Alternatively, drug-bonded hydrogels employ dynamic chemical bonds to reversibly attach anti-infective drugs onto the hydrogel network. Through the design of these dynamic chemical bonds, scientists can not only precisely control the quantity of the drug loaded but also realize sustained or on-demand drug release. Furthermore, by incorporating a range of dynamic chemical bonds or collaborating with drug encapsulation methods, these hydrogels can facilitate the gradual or sequential release of diverse drugs, ensuring optimal delivery and therapeutic effectiveness. During drug release, the hydrogel network in drug-loaded dynamic hydrogels can be tailored to either retain its structure or degrade, thereby fine-tuning the rate and enhancing the efficiency of drug delivery [36,37].

In contrast, inherent dynamic hydrogels are directly crafted from materials that inherently exhibit antibacterial activity. The primary role of the dynamic covalent bonds within these hydrogels is to preserve their three-dimensional structures and endow them with self-healing or injectable properties. As the constituent materials naturally possess the capacity to suppress or eliminate infections, there is no need to incorporate additional anti-infection agents. Hence, this strategy can greatly reduce the emergence of drug resistance development arising from infections and has, therefore, garnered considerable attention in recent years [38,39].

### 3.1. Strategies for Drug-Loaded Dynamic Hydrogels

Anti-infection agents come in many forms, such as small-molecule drugs [40], natural phenolic compounds [41], peptides/proteins [42], and nanoparticles [43]. Each type has its own distinct properties. When designing drug-loaded dynamic hydrogels, it is crucial to consider how to utilize dynamic chemical bonds to efficiently encapsulate and controlled-release these agents, meeting diverse therapeutic requirements [44,45].

**Figure 3:**
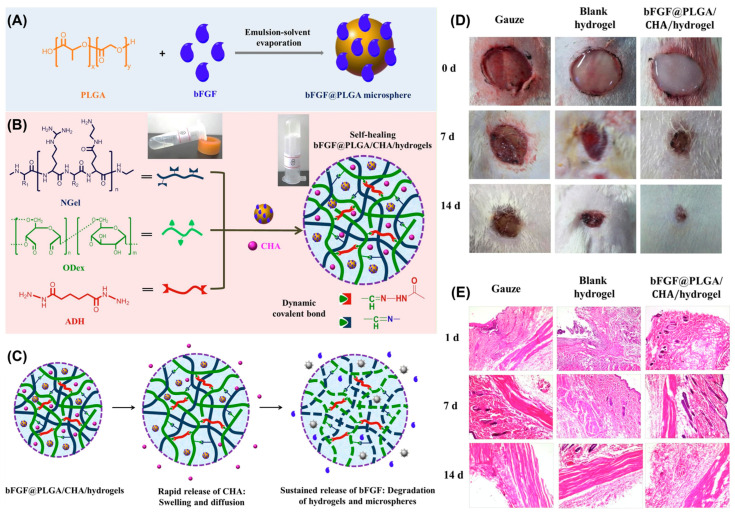
(**A**) Fabrication of poly (lactic-co-glycolic acid) microspheres-loaded bFGF@PLGA microspheres. (**B**) Preparation of bFGF@PLGA/CHA/hydrogels. (**C**) The sequential delivery of bFGF@PLGA/CHA/hydrogels. (**D**) Gross observation and (**E**) H&E staining of the wound skin treated with the gauze, blank hydrogels, and bFGF@PLGA/CHA/hydrogels. Adapted with permission from Ref. [48]. Copyright © 2019 Elsevier B.V.

Small-molecule anti-infection agents are typically chemically synthesized and possess a well-defined structure [46]. They can interact with specific biological targets, inhibiting the growth or replication of microorganisms or viruses. These agents can be either water-soluble or insoluble. For water-soluble small-molecule anti-infection agents, encapsulation and rapid release can be directly achieved using hydrogels. For instance, chlorhexidine acetate (CHA) is a broad-spectrum, highly effective, and low-toxicity anti-infection drug with good water solubility. It exhibits good therapeutic effects against bacteria, particularly Gram-positive bacteria, while also being effective against some fungi and viruses [47]. Liu and Zhang et al. prepared a self-healable hydrogel that can sequentially release CHA and basic fibroblast growth factor (bFGF) (Figure 3A) [48]. This hydrogel was formed through dynamic imine bonds and acylhydrazone bonds among amidated gelatin (NGel), adipic acid dihydrazide (ADH), and oxidized dextran (ODex). Using this hydrogel, they simultaneously encapsulated both CHA and poly (lactic-co-glycolic acid) microspheres loaded with bFGF (bFGF@PLGA) (Figure 3B). During the drug release process, CHA, which was directly dispersed in the hydrogel, was rapidly released to the wound. Meanwhile, the growth factors were gradually released owing to the swelling and degradation properties of hydrogels and PLGA microspheres (Figure 3C). This sequential release ensures that CHA effectively treats infections during the early stages, while the growth factors promote cell proliferation and tissue regeneration in the later stages. Compared with blank hydrogel, the bFGF@PLGA/CHA/hydrogels showed a faster wound contraction rate during treatment, and there was almost no obvious wound area at 14 days (Figure 3D). The H&E results also revealed collagen fiber hyperplasia in the hydrogel group, with many fibroblast cells observed and a noticeable reduction in inflammation after 14 days (Figure 3E). This strategy allows for the adjustment of the release sequence and rate of drugs, offering new possibilities for wound healing.

**Figure 4:**
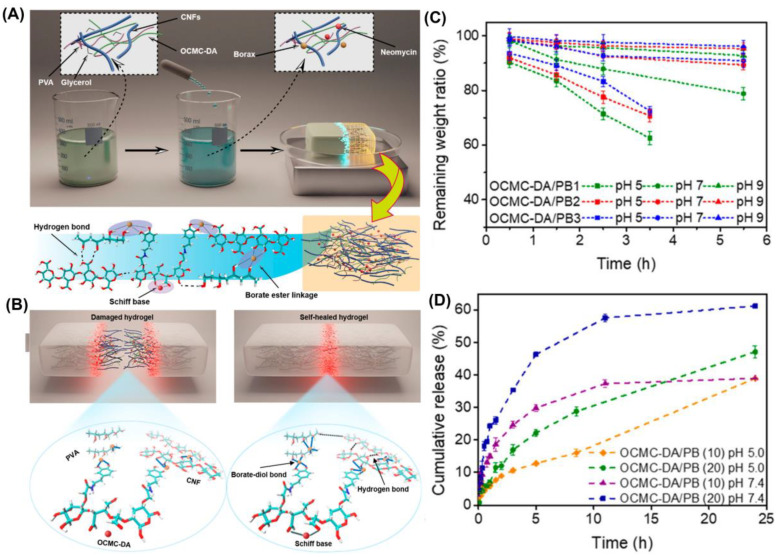
(**A**) Schematic illustration and microscopic structure of OCMC-DA/PB hydrogel; (**B**) The dynamic reversible boronic ester linkages and hydrogen bonds along with the imine bond within the hydrogel. (**C**) The pH-responsive degradability of OCMC-DA/PB hydrogels. (**D**) Drug release behaviors of OCMC-DA/PB hydrogels. Adapted with permission from Ref. [25]. Copyright © 2021 American Chemical Society.

Neomycin (NEO) is a polyaminoglycoside antibiotic noted for its excellent water solubility and potent antibacterial activity against both Gram-positive and Gram-negative bacteria [49]. In a study led by Xiao, Jin and Seidi, a pH-responsive self-healing polyvinyl alcohol (PVA)-borax hydrogel was prepared with the addition of NEO, dopamine-grafted oxidized carboxymethyl cellulose (OCMC-DA), and cellulose nanofibers (CNF) (Figure 4A,B) [25]. In this hydrogel, the polyamines of NEO functioned as extra crosslinkers, reacting with the aldehyde groups present on OCMC-DA to form dynamic imine bonds. These dynamic bonds, combined with the hydrogen bonds and diol-boronic ester bonds that exist between PVA, CNF, and OCMC-DA, enhance the mechanical properties of the hydrogel. Interestingly, despite the expected rapid degradation of these imine bonds under acidic conditions (Figure 4C), experiments revealed that NEO release was relatively slower at pH 5.0 compared to higher pH values. The authors suggested that this was attributed to the protonated state of NEO at this pH, leading to the formation of a polycationic oligosaccharide that strengthens electrostatic interactions with OCMC-DA and boric acid diol anions, thus resulting in sustained drug release (Figure 4D).

Clotrimazole is a drug with broad antifungal properties, yet its poor water solubility hinders its application in the medical field [50]. To address this issue, Gosecka et al. prepared a unimolecular micelle based on hyperbranched polyglycidol (HbPGL) as a drug carrier for clotrimazole and further constructed an injectable hydrogel system via dynamic boronic ester bonds [51]. In this design, the hydrophobic core of the HbPGL unimolecular micelles efficiently encapsulated clotrimazole molecules, while the hydroxyl groups at their shell underwent dynamic crosslinking with boronic acids, anchoring them within the hydrogel network. This design not only ensures the stability of the hydrogel at body temperature but also allows for its rupture and reconstitution in the presence of glucose, enabling effective drug release and imparting self-healing properties to the hydrogel. In this study, the authors also found that the encapsulation efficiency of clotrimazole increases with the degree of hydrophobization (optimized at around 30 mol %) of the HbPGL core modification. Moreover, HbPGL hydrophobized via urethane bonds exhibited a lower rate of clotrimazole release compared to those hydrophobized via ester linkages, facilitating more sustained drug release. This work provides new insights into the controlled release of hydrophobic drugs.

**Figure 5:**
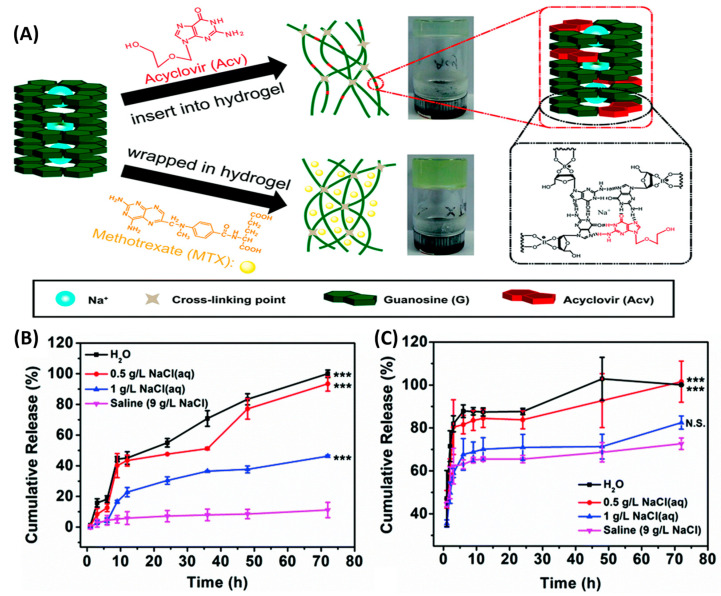
(**A**) Schematic illustration of different drug (methotrexate (MTX) and Acyclovir (Acv)) encapsulation forms in the GB hydrogel. Cumulative (**B**) Acv and (**C**) MTX release as a function of time after exposure to saline (purple line), 1 g L^−1^ NaCl solution (blue line), 0.5 g L^−1^ NaCl solution (red line), and H_2_O (black line). N.S. *p* > 0.05 and *** *p* < 0.001. Adapted with permission from Ref. [53]. Copyright © 2020 Royal Society of Chemistry.

Acyclovir is a small molecule drug mainly used for various infections caused by herpes simplex virus [52]. As it is a derivative of guanosine with the ability to react with boronic salts, Zhang et al. utilized this property to incorporate acyclovir into a dynamic hydrogel system for antiviral therapy [53]. The addition of NaOH into a solution of guanosine (G) and B(OH)_3_ produced the guanosine–borate diesters (GB diesters), which further self-assembled into a G_4_-quartet. The G_4_-quartet was then stacked to form G-nanofibers through π-π stacking interactions, and the lateral cis-diols on the G-nanofibers intertwined by borate ester bonds, eventually inducing the hydrogelation. When acyclovir was introduced, it was able to partially replace the guanosine in the G_4_-quartet, thereby forming a stable encapsulation (Figure 5A). Hence, compared with the physical loading of a non-nucleoside analog methotrexate (MTX) into a hydrogel network, the Acyclovir hydrogel allowed for on-demand release in response to external stimuli such asthe pH, glucose, and cation concentration. As shown in Figure 5B,C, acyclovir and MTX encapsulated in hydrogels exhibited different release profiles in a sodium chloride solution. When the ACV-loaded hydrogel was immersed in a 1 g/L NaCl aqueous solution, only 40% of ACV was released, while the MTX group released nearly 80%. Furthermore, as the concentration of sodium ions increased, the release rate of acyclovir was further reduced. These data demonstrate that the integration of drugs into the hydrogel network through dynamic covalent bonds can effectively enhance the efficacy of antimicrobials or antivirals, providing a new solution for addressing the issue of the reduced therapeutic effect caused by initial drug burst release.

**Figure 6:**
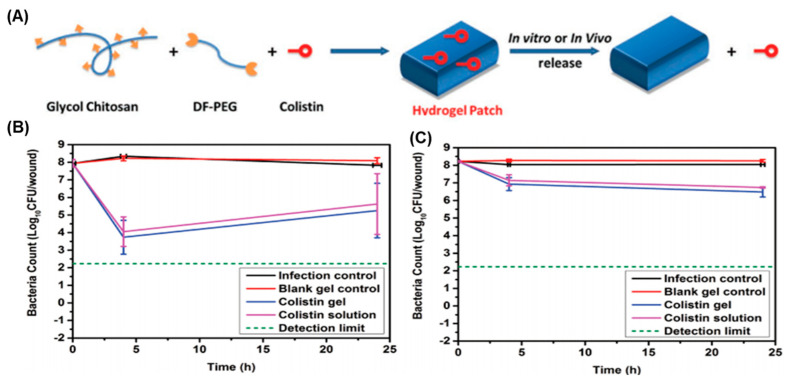
(**A**) Illustration of the synthesis of the colistin-containing hydrogel. The “burn” infection model test of the colistin-loaded hydrogel against (**B**) colistin-sensitive and (**C**) colistin-resistant strains. Adapted with permission from Ref. [56]. Copyright © 2016 Wiley-VCH.

Some peptides, proteins, and small molecular compounds produced by bacteria, insects, plants, and animals also show potent anti-infection properties [54]. However, due to their potential toxicity, many of them are not suitable for direct clinical use. Incorporating these peptides and proteins into hydrogels has emerged as a successful strategy for preserving their antimicrobial activity while minimizing their toxicity. For instance, colistin (polymyxin E) is a lipopeptide antibiotic capable of specifically binding to the lipopolysaccharide of the outer membrane of Gram-negative bacteria, thereby inducing cell death [55]. However, its potential nephrotoxicity, as well as its sensitivity to thermal, enzymatic, and chemical conditions, hinders its use for systemic administration. Therefore, Velkov, Haddleton, and their colleagues incorporated colistin into a hydrogel with dynamic imine bonds by reacting amino-rich glycol chitosan with a biocompatible aldehyde-modified polyethylene glycol crosslinker (DF-PEG) (Figure 6A) [56]. Interestingly, the authors found that the addition of colistin not only provided anti-infection properties but also accelerated the gelling process of this hydrogel. In this system, the water-soluble colistin was uniformly distributed within the hydrogel and can achieve a high loading capacity. As a result, this colistin-loaded hydrogel was able to kill not only colistin-sensitive strains of *P. aeruginosa* (Figure 6B) but also colistin-resistant “superbugs” (Figure 6C) in vivo within a mouse burn infection model. Thanks to the biodegradable and dynamic nature of the hydrogel, this material exhibited “on-wound” degradation in response to the local wound environment, offering a viable treatment for “superbug” wound infections. 

Actinomycin X2 (Ac.X2), derived from Streptomyces cyaneofuscatus fermentation, is a cyclic antimicrobial peptide effective against Gram-positive bacteria [57]. To reduce its cytotoxicity, Han et al. developed an antimicrobial dynamic hydrogel by integrating PVA, CMCS, protocatechualdehyde (PA), ferric ions (Fe^3+^), and Ac.X2. Strengthened by hydrogen bonding and double dynamic crosslinkings [58], this hydrogel exhibits self-healing performances and improved mechanical properties, allowing it to be compressed and bent without any fracture. Moreover, thanks to the antimicrobial activity of CMCS, this hydrogel is not only effective against Gram-positive bacteria but also demonstrates good activity against Gram-negative bacteria, thereby broadening the potential applications of Ac.X2.

Nanoparticles are receiving increasing attention in the fields of antibacterial and antiviral applications due to their unique size effect, catalytic activity, etc. Taking silver nanoparticles (AgNPs) as an example, they are believed to disrupt the mitochondrial respiratory chain of bacteria, leading to the production of reactive oxygen species and bacteria death. Some studies have also found that AgNPs can bind to envelope proteins, preventing viruses from entering cells and preventing virion infectivity [59]. Other inorganic nanoparticles, such as zinc oxide [60], titanium dioxide [61], and Mxene [62], have also demonstrated excellent inhibition against microorganisms and viruses through various mechanisms. Directly incorporating these nanoparticles into dynamic hydrogels allows for producing self-healable wound dressings and injectable formulations for anti-infection treatment.

**Figure 7:**
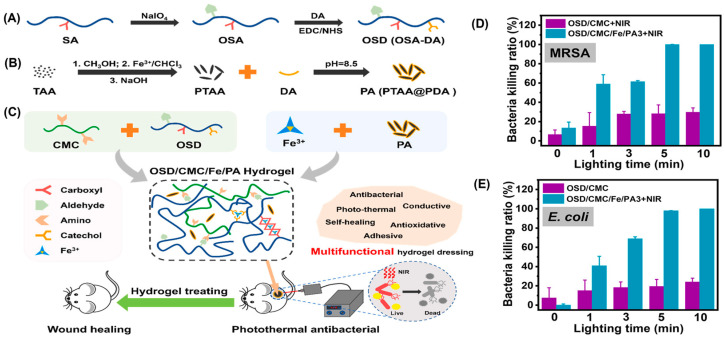
(**A**) Preparation scheme of oxidized sodium alginate-grafted dopamine (OSD) and (**B**) polydopamine-coating poly (thiophene-3-acetic acid) (PA); (**C**) Preparation and application of OSD/carboxymethyl chitosan/Fe^3+^/PA (OSD/CMC/Fe/PA) hydrogels. The killing ratio of (**D**) MRSA and (**E**) *E. coli* in OSD/CMC and OSD/CMC/Fe/PA3 hydrogels under NIR with different lighting times. Adapted with permission from Ref. [65]. Copyright © 2023 Elsevier B.V.

In addition to inorganic nanoparticles, organic nanoparticles can also be used for anti-infection treatment. For instance, poly (thiophene-3-acetic acid) (PTAA) is a photothermal agent with good biocompatibility and chemical stability [63,64]. Guo et al. modified PTAA with polydopamine to obtain a water-soluble photothermal nanoparticle (PA) and utilized it to fabricate a dual dynamic bond crosslinked hydrogel with photothermal antibacterial properties under near-infrared (NIR) light irradiation [65]. The hydrogel consisted of dopamine-modified oxidized sodium alginate (OSD), carboxymethyl chitosan (CMC), Fe^3+^, and PA, in which the aldehyde groups on OSD react with amines on CMC to form dynamic imine bonds as the primary crosslinking, while Fe^3+^ coordinates with carboxyl groups in CMC and catechol groups in dopamine to form the secondary dynamic crosslinking (Figure 7A–C). Due to the dynamic network, this hydrogel is self-healable, enabling the dressing to seamlessly adapt to complex wound surfaces. The prepared hydrogel (with 3 wt% PA, namely, OSD/CMC/Fe/PA3) killed over 99% of MRSA within about 5 min of NIR light exposure (Figure 7D), while it killed all *E. coli* within 10 min (Figure 7E). Furthermore, these hydrogels exhibited exceptional conductivity, tunable rheology, suitable mechanical strength, antioxidant activity, tissue adhesion, and hemostatic properties. These combined features make this hydrogel a promising candidate for the treatment of wound infections.

### 3.2. Hydrogels with Inherent Anti-Infection Performance

Bacterial cell membranes and airborne fungal spores typically carry negative charges, while many viruses also exhibit negatively charged surfaces in aqueous solutions [66]. Therefore, cationic compounds can effectively bind to these pathogens, inhibiting their growth or killing them [67]. Among these compounds, chitosan, which is derived from chitin and is abundant in amino groups, has been widely applied in the construction of hydrogels with antimicrobial activity. The amino groups in chitosan-based hydrogels become positively charged under physiological conditions, enabling them to disrupt bacterial cell walls. Thus, these hydrogels exhibit an inherent anti-infection performance even without additional antimicrobial agents [68]. Recently, dynamic chemical bonds have been successfully installed into these hydrogels, further broadening their applications.

Despite the widespread availability, low cost, and excellent biocompatibility of chitosan, its limited solubility in physiological pH restricts its application in the medical field. To improve the solubility of chitosan, researchers have developed modified chitosan derivatives, including quaternized chitosan, hydroxypropyl chitosan, hydroxyethyl chitosan, carboxymethyl chitosan, etc., and have utilized them for the fabrication of dynamic hydrogels [69]. For example, Li’s group prepared a dynamic antibacterial hydrogel through the reaction between quaternized chitosan (QCS) and aromatic aldehyde-functionalized polyethylene glycol diacrylate (PEGDA) [70]. Owing to the presence of a dynamic imine bond, this hydrogel exhibited adaptive and self-healing performances. Furthermore, the cationic groups of this hydrogel not only confer moderate adhesive properties but also interact with bacterial cell membranes, thereby demonstrating significant antibacterial activity against both Gram-positive (*S. aureus*) and Gram-negative (*E. coli*) bacteria. Additionally, its antibiotic-free nature ensures that its cationic antibacterial action does not induce bacterial resistance and can maintain a prolonged antibacterial effect. 

**Figure 8:**
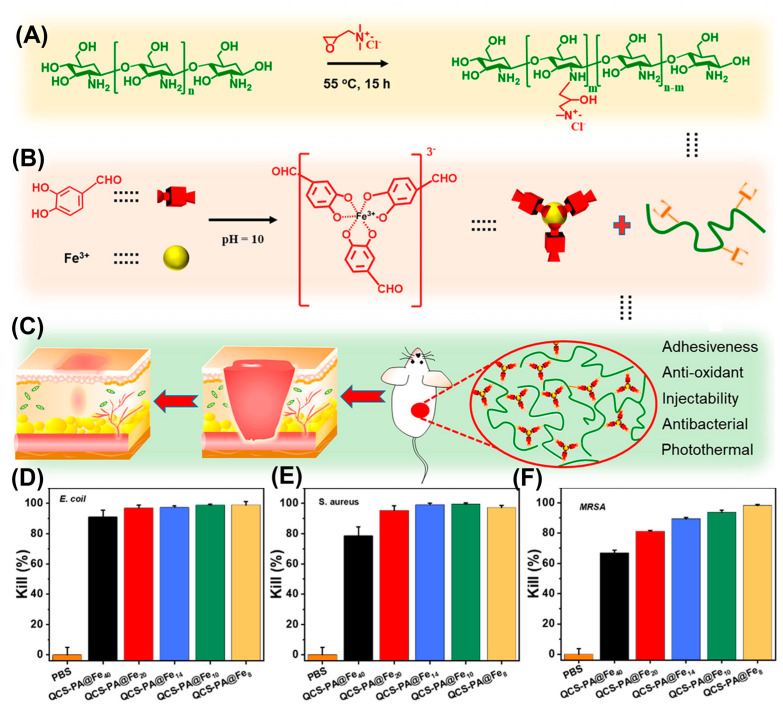
(**A**) Synthesis of quaternized chitosan. The molar ratio of GTMAC to amino groups on the chitosan backbone was set as 2:1. (**B**) Fabrication of a PA@Fe tricomplex molecule (pH = 10). (**C**) Dual-dynamic-bond cross-linked adhesive hydrogel shows applications in wound closure and post-wound-closure care. Quantitative results of the surface antibacterial properties of the hydrogels (the molar ratio of the amino group and aldehyde group increased from 40:1 to 8:1) against (**D**) *E. coli*, (**E**) *S. aureus*, and (**F**) MRSA. Adapted with permission from Ref. [71]. Copyright © 2021 American Chemical Society.

Compared to the introduction of a single type of dynamic chemical bonds in the previous example, Guo et al. utilized protocatechualdehyde to construct a dual-dynamic-bond cross-linked quaternized chitosan (QCS) hydrogel [71]. The catechol group on protocatechualdehyde formed a coordination complex with Fe^3+^ (PA@Fe), while the aldehyde group on protocatechualdehyde underwent a Schiff base reaction with the amino group of QCS (Figure 8A,B). This dual-dynamic-bond cross-linking endows the hydrogel with a pH response and good mechanical strength (Figure 8C). Through the antibacterial tests, the authors demonstrated that this hydrogel exhibited excellent antimicrobial activity against both Gram-negative (*E. coli*) (Figure 8D) and Gram-positive (*S. aureus*) (Figure 8E) strains due to the inherent antibacterial performance of QCS. Moreover, as the content of PA@Fe increased, this hydrogel was able to kill methicillin-resistant *S. aureus* (MRSA) with a high efficiency, which was attributed to the synergetic antibacterial activity of PA@Fe and QCS (Figure 8F). Under NIR irradiation, PA@Fe in this hydrogel served as photothermal agents, producing a controlled photothermal effect to denature the enzymes in the bacteria, giving a high in vivo antimicrobial activity against MRSA. This work demonstrates the potential of dynamic hydrogels with inherent antimicrobial activity as wound dressings for drug-resistant pathogens.

## 4. Challenges and Prospects

Despite the advantages of dynamic hydrogels in anti-infection treatments, they still encounter numerous challenges in practical applications. For instance, the introduction of dynamic bonds into hydrogels may complicate the synthesis process and increase costs, while also compromising their mechanical properties, stability, and biocompatibility. Hence, the continuous development of new chemical bonds, as well as new strategies for hydrogel design, are still desired. With the new emerging chemical bonds, we may incorporate more useful anti-infection agents in their original forms, enhancing the drug encapsulation efficiency without introducing additional functional groups. By combining various dynamic bonds, we can also achieve multi-step drug administration or synergize with other anti-infection mechanisms such as photothermal treatment, resulting in a more complex and effective anti-infection approach to tackling complicated infections and reducing the emergence of drug resistance. 

In addition, the current morphologies of dynamic hydrogels are primarily limited to bulk materials. It is imperative to shape them into more diverse structures to further broaden their potential in anti-infection applications. Fortunately, with the advent of novel processing techniques, this design objective is now within reach. Despite the use of non-dynamically responsive gelatin hydrogels, Liu et al. demonstrated the potential of employing 3D printing technology to fabricate antibacterial rigid hydrogels as meniscus substitutes, providing evidence that antimicrobial hydrogels with specific structures are beneficial in reducing implant failures caused by infections [72]. Therefore, it is foreseen that the combination of 3D printing and dynamic hydrogels may be a powerful tool in addressing the anti-infection treatment requirements of various bodily regions while simultaneously offering self-healing and injectable properties. 

Furthermore, dynamic hydrogels exhibit a remarkable ability to adjust their inherent physical and chemical properties in response to external stimuli, such as temperature, pH, or specific biomolecules. This adaptability, coupled with their unique traits like self-healing, positions them as highly advantageous in the treatment of infections. However, due to the dynamic characteristics of these hydrogels, their mechanical strength is usually lower than that of traditional hydrogels, limiting their potential applications beyond wound dressings. Although the introduction of a fixed crosslinking network is expected to enhance the strength of these hydrogels, it may weaken their original dynamic properties. Recent studies have shown that methods such as adopting a dynamic dual-network structure can achieve a balance between enhancing mechanical strength and maintaining the original characteristics of the hydrogels [73]. Nevertheless, related research is still in its early stages. Therefore, how to customize the mechanical properties and dynamics of dynamic gels according to specific needs has become another important research direction.

## 5. Conclusions

In summary, we have demonstrated the unique responsive behaviors of dynamic hydrogels and highlighted their significant advantages in the field of anti-infection. Compared to traditional hydrogels, dynamic hydrogels exhibit superior adaptability, allowing them to conform more closely to wound contours and form a robust barrier against external invaders. Furthermore, the self-healing performance of these dynamic hydrogels allows them to swiftly restore their mechanical properties and maintain continuous protection for the wound even after sustaining damage from external forces. Thanks to their dynamic networks, the dynamic hydrogels can be easily formulated into injectable forms, providing alternative administration approaches. Since the application of dynamic hydrogel in anti-infections is still in the early stage of research, there are few clinical data and related patents at present, not to mention the products on the market. We eagerly await the emergence of innovative chemical bond varieties and complex morphology designs of these hydrogels to deliver even more effectiveness and secure therapeutic options for treating infections. It is expected that dynamic hydrogels will play a more important role in the future medical field and make greater contributions to human health.

## Data Availability

Not applicable.
